# Decreased Phosphorylation and Increased Methionine Oxidation of *α*-Synuclein in the Methionine Sulfoxide Reductase A Knockout Mouse

**DOI:** 10.4061/2011/721094

**Published:** 2011-01-12

**Authors:** Derek B. Oien, Gonzalo A. Carrasco, Jackob Moskovitz

**Affiliations:** Department of Pharmacology and Toxicology, School of Pharmacy, The University of Kansas, Lawrence, KS 66045, USA

## Abstract

Previously, we have showed that overexpression of methionine-oxidized *α*-synuclein in methionine sulfoxide reductase A (MsrA) null mutant yeast cells inhibits *α*-synuclein phosphorylation and increases protein fibrillation. The current studies show that ablation of mouse *MsrA* gene caused enhanced methionine oxidation of *α*-synuclein while reducing its own phophorylation levels, especially in the hydrophobic cell-extracted fraction. These data provide supportive evidence that a compromised MsrA function in mammalian brain may cause enhanced pathologies associated with altered *α*-synuclein oxidation and phosphorylation levels.

## 1. Introduction

Parkinson's disease (PD) is characterized by the formation of neuronal inclusion bodies that are denoted as Lewy bodies [1]. These bodies consist mainly of *α*-synuclein fibrils [2]. The *α*-synuclein protein is a presynaptic and its function and involvement in the development of PD is yet to be clearly determined. When *α*-synuclein is abnormally expressed or modified, various studies indicate it may cause alterations in mitochondrial and proteasomal function, protein aggregation, and accumulation of reactive oxygen species (ROS) [3–6]. 

Increased ROS and cellular *α*-synuclein levels promote its aggregation [7, 8] and can cause posttranslational modifications to the methionine (Met) residues of *α*-synuclein leading to the formation of methionine sulfoxide (MetO)—containing *α*-synuclein (MetO-*α*-synuclein). MetO modifications can be present in two forms of enantiomers: Met-*S*-O and Met-*R*-O that can be readily reduced by the methionine sulfoxide reductase (Msr) system. Msr type A (*MsrA*) reduces Met-*S*-O and Msr type B (MsrB) reduces Met-*R*-O [9, 10]. *MsrA* is thought to be the major Msr because it is a positive regulator of MsrB expression levels [11, 12]. Absence of *MsrA* can cause a hypervulnerability to conditions of oxidative stress [13–17] and shortened lifespan [16, 18]. One possible explanation is that the cellular regulation of aging neurons (e.g., in PD) is altered and consequently causes the accumulation of misfolded proteins (e.g., *α*-synuclein) [19]. 

The functional role of phosphorylation to *α*-synuclein is not completely and clearly understood. Previous work has determined that *msrA* knockout yeast strain expressing either of the three *α*-synuclein types (normal type *α*-synuclein (Syn) and Syn mutant types: SynA30P, SynA53T) showed significantly lower levels of phosphorylation relative to *α*-synuclein expressed in wild-type cells [22]. These data suggest that increased levels of MetO moiety in *α*-synuclein could inhibit its phosphorylation efficiency, especially when expressed in *msrA*-KO cells. Supportive evidence for such possibility was demonstrated by a reduced *in vitro* phosphorylation of these three types of *α*-synuclein by casein kinase 2 (a major kinase for *α*-synuclein Ser^129^ phosphorylation [21]) following oxidation of *α*-synuclein [22]. 

The observed decrease in *α*-synuclein phosphorylation after MetO formation when *MsrA* is ablated in yeast cells may mimic processes that regulate *α*-synuclein phosphorylation in mammals. Further investigations in a parallel mammalian system will greatly contribute to clarify the physiological role of *MsrA* on *α*-synuclein phosphorylation. Accordingly, the goal of the current studies is to determine the levels of MetO and *α*-synuclein phosphorylation in brains of *MsrA* knockout (*MsrA* ^−/−^) and WT mice and evaluate their intracorrelation.

## 2. Materials and Methods

### 2.1. Materials

[*γ*-^32^P]-ATP (3,000 Ci/mmol) was purchased from Perkin-Elmer (Waltham, MA) and casein kinase 2 was purchased from Millipore (Billerica, MA). Rabbit anti-MetO antibodies were made in our lab [20] and antibodies against *α*-synuclein were purchased from BD.

### 2.2. Methods

#### 2.2.1. Phosphorylation of Proteins in Brains of WT and MsrA^−/−^ Mouse Brains

**Protein Extraction**

Postmortem brains of each mouse types (6 months of age) were homogenized at 4°C in the presence of 25 mM Tris (pH 7.4) and protease inhibitor cocktail (no-EDTA) (Roche, South San Francisco, CA) and 1 mM CaCl_2_. The extracted protein fractions were centrifuged (15,000 × g) for 20 minutes at 4°C, and the resulting Tris-soluble supernatants (Sup 1) were kept and stored at −80°C. The pellets obtained following the centrifugation step were redissolved in 25 mM Tris (pH 7.4) and 48% Urea. Then, the urea-soluble pellets were centrifuged again as described above, and the resulting supernatants were dialyzed against 25 mM Tris (pH 7.4) at 4°C (Sup 2).

#### 2.2.2. Phosphorylation Assay

Fifty microgram of Sup 1 and a mixture containing 40 *μ*g of Sup 2 and 10 *μ*g of Sup 1 (serving as a source for kinases) were incubated in the presence of 25 mM Tris (pH 7.4), protease inhibitor cocktail (no-EDTA) (Roche, South San Francisco, CA), 1 mM CaCl_2_, 10 mM MgCl_2_, and 16.7 *μ*M [*γ*-^32^P]-ATP (3000 Ci/ mmol) for 3 minutes at room temperature in a final volume of 50 *μ*L. Endogenous phosphorylation was stopped by addition of 10 mM EDTA, 10 mM EGTA, and 1 mM cold ATP and immediately placed on ice. Then, the samples were subjected to an immunoprecipitation by anti-*α*-synuclein antibodies or anti-MetO antibodies as described below.

#### 2.2.3. Isolation of Phosphorylated *α*-Synuclein from Brain Extracts

Postmortem brains (*n* = 5) of each mouse types were processed according to the protocol provided by the Phosphoprotein Purification Kit purchased from Qiagen (Valencia, CA). Briefly, each brain tissue was homogenized using 0.25% (w/v) CHAPS solution in lysis buffer containing protease inhibitor cocktail and Benzonase. Following incubation at 4°C for 30 min, homogenates were centrifuged at 10,000 × g and 4°C for 30 min. Protein concentrations of the corresponding supernatants were determined by Coomassie (Bradford) Protein Assay Kit (Thermo Fisher Scientific, Rockford, IL), and 2.5 mg of total protein of each lysate was applied into the Phosphoprotein Purification column according to the protocol instructions. Then, the unbound proteins were washed out from the column and the column-bound proteins were eluted with the elution buffer and concentrated using centricon with molecular cutoff of 3 kDa (Millipore, Billerica, MA). Finally, equal protein amounts (50 *μ*g) of each concentrated sample were subjected to an SDS-PAGE (4%–20%) separation followed by Western blot analysis using primary antibodies against *α*-synuclein (BD, Franklin Lakes, NJ).

#### 2.2.4. Immunoprecipitation or Western Blot (IP) Assays

Immunoprecipitation was performed on brain soluble fractions described above (Sup1 and Sup2) by using primary antibodies against either *α*-synuclein (BD, Franklin Lakes, NJ) or antibodies against MetO [20]. The immunoprecipitation procedure was followed according to the immunoprecipitation kit's protocol and related supplies provided by Pierce Inc. Thereafter, equal protein amounts of the immunopercipitants were subjected to an SDS-gel electrophoresis (4%–20%) followed by either Western blot analysis using anti-*α*-synuclein antibodies (as primary antibodies) or directly exposed to X-ray film when [*γ*-^32^P]-ATP was used in the phosphorylation assay. The levels of the resulting reacting bands were quantified by densitometry analysis. In the Western blot analysis using the primary anti-*α*-synuclein antibodies, normalization of the *α*-synuclein detected bands in the MetO precipitants was normalized to the levels of *α*-synuclein bands detected directly in each protein extract fraction prior to the immunoprecipitation procedure (50 *μ*g protein per lane).

## 3. Results and Discussion

**Figure 1:**
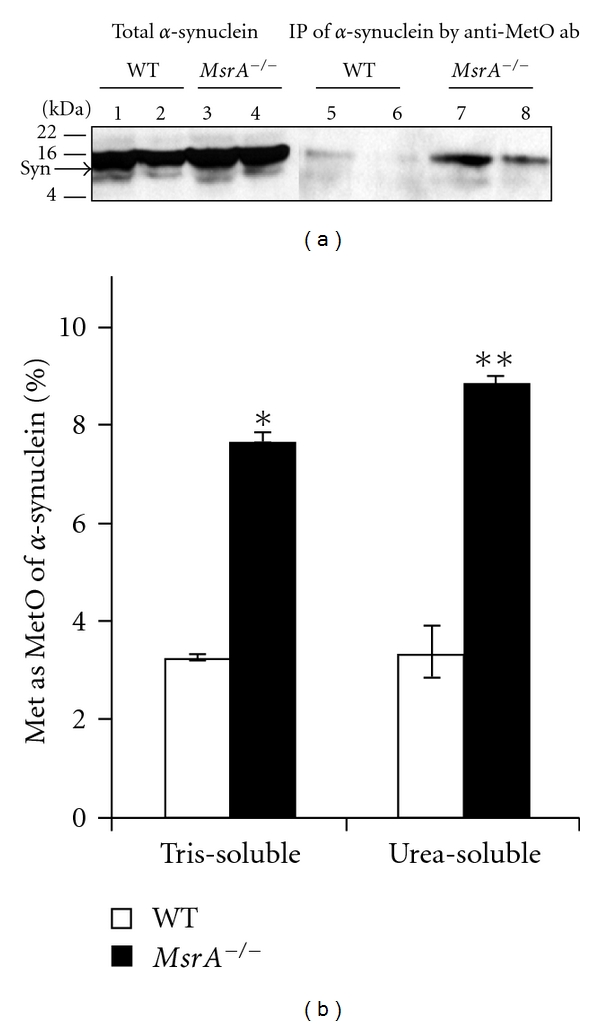
Detection of methionine oxidation of *α*-synuclein in *MsrA* ^−/−^ and wild-type control (WT) brains. (a) Brain protein extracts were prepared as Tris-soluble and urea-soluble fractions from both mouse types. Equal protein amounts of the protein extracts were either directly subjected to SDS-gel electrophoresis and Western blot analysis (Lanes 1–4) or immunoprecipitated by anti-MetO antibodies prior to the gel-loading (Lanes 5–8). Lanes 1, 3, 5, and 7 represent Tris-soluble fractions, and lanes 2, 4, 6, and 8 represent urea-soluble fractions. The Western blot analysis was probed with anti-*α*-synuclein antibodies as primary antibodies. The Western blot analysis is a representative blot of data generated from multiple experiments (*n* = 5). Syn, *α*-synuclein; IP, immunoprecipitation; ab, antibodies; kDA, molecular mass markers in kilo-Dalton. (b) Densitometry analysis of the *α*-synuclein bands detected in (a). The percent MetO values were calculated as percent ratio of the immunoprecipitant MetO-*α*-synuclein bands (Figure 1(a), lanes 5–8) to the corresponding bands representing total *α*-synuclein levels (Figure 1(a), lanes 1–4). The statistical differences between the MetO-*α*-synuclein levels in both mouse types were significant (*n* = 5; *P < .0003 and **P < .003 by *t*-test).

**Figure 2:**
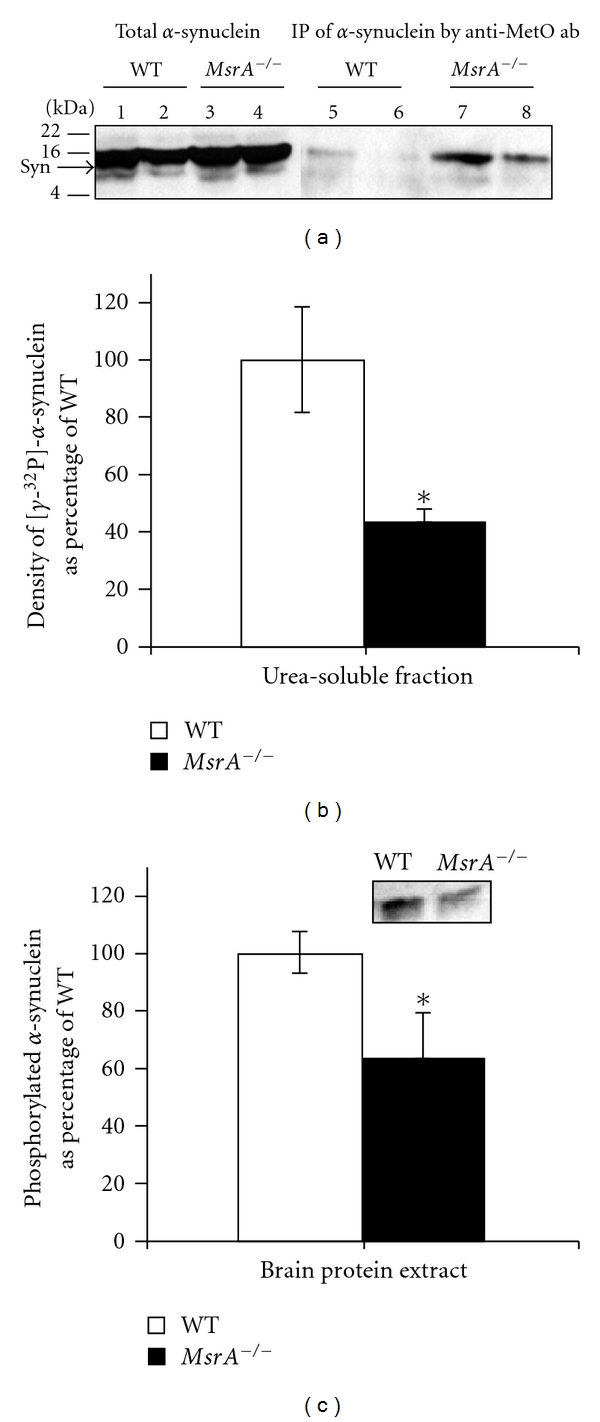
Phosphorylation of *α*-synuclein in *MsrA* ^−/−^ and wild-type (WT) brain extracts. (a) Tris-soluble and Urea-soluble brain extracts (40 *μ*g protein) of both mouse types were prepared as described in Section 2. These extracts were then incubated in the presence of additional brain-matched Tris-soluble extract (10 *μ*g protein, serving as a source for kinases), 25 mM Tris (pH 7.4), protease inhibitor cocktail (no-EDTA) (Roche), 1 mM CaCl_2_, 10 mM MgCl_2_ and 16.7 *μ*M [*γ*-^32^P]-ATP for 3 minutes at room temperature in a final volume of 50 *μ*L. Endogenous phosphorylation was stopped by addition of 10 mM EDTA, 10 mM EGTA, and 1 mM cold ATP and immediately placed on ice. Then, the samples were subjected to an Immunoprecipitation by anti-*α*-synuclein antibodies or anti-MetO antibodies as described in Section 2. Thereafter, equal protein amounts of the immunoprecipitants were subjected to an SDS-gel electrophoresis (4%–20%) followed by exposure of the gel to an X-ray film. Lanes 1, 3, 5, and 7 represent Tris-soluble fractions and lanes 2, 4, 6, and 8 represent Urea-soluble fractions. Syn, *α*-synuclein; ab, antibodies; kDA, molecular mass markers in kilo-Dalton. The detected band following the immunoprecipitation by anti-MetO antibodies was also denoted in the text as MetO-15. (b) Densitometry analysis of authoradiographed bands detected in (a). The percent MetO values were calculated as percent ratio of the detected immunoprecipitant [*γ*-^32^P]-*α*-synuclein in the Urea-soluble fractions, in which WT's [*γ*-^32^P]-*α*-synuclein-averaged value represents 100%. The statistical difference between the [*γ*-^32^P]-*α*-synuclein levels in both mouse types was significant (*n* = 5; *P < .05 by *t*-test). (c) Densitometry analysis of the phosphorylated *α*-synuclein bands detected in Western blot analysis (a representative blot is shown in a small window) after isolation of phosphorylated proteins from WT and *MsrA* ^−/−^ brains (using PhosphoProtein Purification Kit (Qiagen)). See Section 2 for relevant procedures and analyses. The statistical difference between the phosphorylated *α*-synuclein levels in both mouse types was significant (*n* = 5; *P < .05 by *t*-test).

Previously, we have showed that Met oxidation inhibits the phosphorylation of *α*-synuclein both* in vitro* and *in ex vivo* systems, in which *α*-synuclein was overexpressed in yeast [22]. Complementary *MsrA* was able to reduce several oxidized Met residues in recombinant *α*-synuclein [23]. Similarly, studies on other proteins showed that methionine oxidation can inhibit protein phosphorylation [24]. In addition to the phosphorylation inhibition of *α*-synuclein expressed in yeast, general protein degradation and *α*-synuclein degradation were inhibited as well in this *ex vivo* system while increasing protein fibrillation [22]. To establish the relationships between *α*-synuclein phosphorylation and methionine oxidation in a mammalian model, we have monitored first the levels of methionine-oxidized *α*-synuclein in *MsrA* ^−/−^ compared with control wild-type mice. Accordingly, the levels of total *α*-synuclein and MetO-*α*-synuclein were monitored in WT and *MsrA* ^−/−^ brains by Western blot analysis, following immunoprecipitation by the novel anti-MetO antibodies (Figure 1(a)). The presented data clearly show that in comparison to WT, the levels of MetO *α*-synuclein are significantly elevated in *MsrA* ^−/−^ brains both within the Tris-soluble (Sup 1) and urea-soluble (Sup 2) fractions as demonstrated by band densitometry analysis (Figure 1(b)). These results confirm our initial observation showing that the levels of MetO-containing proteins are increased in *MsrA* ^−/−^ brain [25] and in serum proteins as function of age [20]. In addition, the percent of MetO in the analyzed fractions is similar to the *in vivo* MetO percentile observed in yeast cells exposed to oxidative stress [14, 15] (the percent MetO values were calculated as percent ratio of the immunoprecipitated MetO-*α*-synuclein bands (Figure 1(a), lanes 5–8) to the corresponding bands representing total *α*-synuclein levels (Figure 1(a), lanes 1–4)). The next step was to evaluate the effect of the observed methionine oxidation of *α*-synuclein on its phosphorylation levels in brain. To mimic *in vivo* phosphorylation, brain fractions were incubated in a reaction mixture containing [*γ* ^32^P]-ATP followed by immunoprecipitation with anti-*α*-synuclein antibodies or anti-MetO antibodies. Then, the resulting precipitates were subjected to SDS-PAGE and levels of radiolabeled proteins were detected and quantified by exposure to X-ray film. If indeed methionine oxidation interferes with protein phosphorylation, it is expected that levels of phosphorylation detected in the precipitated proteins will be in inverse correlation with the levels of MetO in these proteins. As shown in Figure 2(a), immunoprecipitation of *α*-synuclein resulted in significantly higher levels of phosphorylated *α*-synuclein in the WT's urea-soluble fraction compared with the corresponding *MsrA* ^−/−^ fraction. Interestingly, phosphorylated *α*-synuclein was detected only in the urea-soluble fraction and not detected in the Tris-soluble fraction (Figure 2(a), lanes 2 and 4). These results confirm our initial observation in the *ex vivo* system, in which the phosphorylation of recombinant *α*-synuclein was inhibited in *msrA* null mutant yeast cells [22]. Moreover, the demonstrated enhanced protein fibrillation in *α*-synuclein-expressing *msrA* knockout yeast cells may relate to the lower *α*-synuclein phosphorylation in membranal/insoluble* MsrA* ^−/−^ fraction, as shown here in mouse brain (Figure 2(a)). The role of phosphorylation of *α*-synuclein is controversial. *In vitro *phosphorylation of *α*-synuclein at Ser^129^ by casein kinase 2 forms fibrils faster than nonphosphorylated *α*-synuclein [26] and fosters the formation of cytoplasmic inclusion bodies in cell culture models of synucleinopathies. However, *in vivo* studies showed no obvious correlation between phosphorylated Ser^129^ and *α*-synuclein fibril formation [27]. In contrast, another study demonstrated that phosphorylation at Ser^129^ inhibits *α*-synuclein fibril formation *in vitro* [28]. 

In light of the recent and current data, it is intriguing to propose that phosphorylation of *α*-synuclein may be a cellular compensatory attempt aimed at increasing its solubility and preventing its aggregation. This suggested role of *α*-synuclein phopshorylation is especially important under conditions of oxidative stress that lead to enhanced methionine oxidation. Typically, protein oxidation will cause a Met residue to be more hydrophilic, but the overall MetO protein may become more hydrophobic by exposing hidden hydrophobic residues [29]. Therefore, enhanced formation of MetO-*α*-synuclein (while causing its reduced phosphorylation rate) is expected to increase its hydrophobic properties and as a consequence of its aggregation. Immunoprecipitation of [*γ*-^32^P]-labeled proteins by anti-MetO antibodies revealed a distinct protein band with a similar molecular mass of *α*-synuclein (Figure 2(a), lanes 6 and 8). To facilitate discussion, this protein band will be denoted as MetO-15. Like shown with the [*γ*-^32^P]-labeled *α*-synuclein precipitants (Figure 2(a)), the relative intensity of this band was greater in the WT's urea-soluble fraction than its parallel *MsrA* ^−/−^ fraction. Although the WT fraction contains MetO proteins (as evident by the performed MetO-protein Immunoprecipitation), the MetO residue levels in the WT fraction are lower compared with *MsrA* ^−/−^ fraction, and thereby causing higher phosphorylation level of the detected WT's MetO-15 (Figure 2(a)). The identification of this MetO-15 band by mass spectrometry was not possible due to limits of detection. However, by its molecular mass, it seems that this protein may be either MetO-*α*-synuclein or a protein with a similar molecular mass as previously observed [25]. Regardless of MetO-15 identity, the phosphorylation levels of this protein provide a good indication on the MetO effect on protein phosphorylation in each mouse type. To better relate to the levels of brain *α*-synuclein phosphorylation in each mouse type, densitometry analyses of the phosphorylated *α*-synuclein bands were performed. The averaged value calculated for the WT brain represented a 100% phosphorylation rate (Figure 2(b)). Accordingly, the averaged level of phosphorylated *α*-synuclein in the *MsrA* ^−/−^ Urea-soluble fraction was inhibited by 60% in comparison to the WT's Urea-soluble fraction (Figure 2(b)). This MetO-mediated inhibition of *α*-synuclein phosphorylation through *MsrA* gene ablation is in agreement with the presence of significantly higher levels of *in vivo* methionine oxidation of *α*-synuclein in *MsrA* ^−/−^ brain (Figure 1). Additional supportive evidence for this phenomenon is the observation showing that *in vivo* phosphorylation of *α*-synuclein is lower by 36% in *MsrA* ^−/−^ compared with WT brain (Figure 2(c)). It is suggested that a compromised *MsrA* function may cause alteration of *α*-synuclein's methionine sulfoxide levels that in turn affect *α*-synuclein's phosphorylation levels. Consequently, the ratio of *α*-synuclein's phosphorylated form to its nonphosphorylated form may contribute to the progression of neurodegenerative diseases like PD. 

Other *in vitro* effects of methionine oxidation on *α*-synuclein have been extensively described in the literature. For example, the role of methionine oxidation on *α*-synuclein fibrillation has been studied *in vitro,* and it seems that MetO inhibits *α*-synuclein fibrillation, depending on the environmental conditions [30–35]. In addition, it was reported that MetO may affect dopamine binding to *α*-synuclein [36, 37] and its ability to carry out one of its proposed functions as an antioxidant [38].

 In summary, it is possible that *α*-synuclein structure and function may be regulated through *in vivo* oxidation of its Met residues and their reversal reduction by the Msr system.

Further investigations are underway to determine if the observed *in vitro* effects of MetO on *α*-synuclein's structure and function can also be detected *in vivo*.
