# Erratum to “Decreased Phosphorylation and Increased Methionine Oxidation of **α**-Synuclein in the Methionine Sulfoxide Reductase A Knockout Mouse”

**DOI:** 10.1155/2012/415713

**Published:** 2012-12-20

**Authors:** Derek B. Oien, Gonzalo A. Carrasco, Jackob Moskovitz

**Affiliations:** Department of Pharmacology and Toxicology, School of Pharmacy, University of Kansas, Lawrence, KS 66045, USA

On page 4 of this published article an error in Figure 2(a) has occurred. Accordingly, Figure 2(a) has been replaced with a corrected version of the figure as shown below.

## Figures and Tables

**Figure 2:**
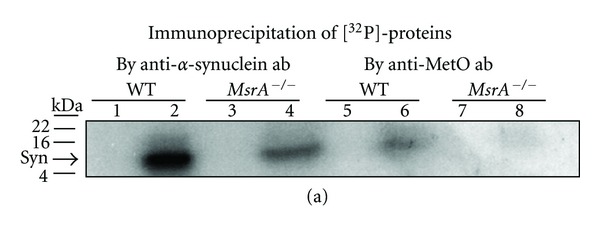
Phosphorylation of *α*-synuclein in *MsrA* ^−/−^ and wild-type (WT) brain extracts. (a) Tris-soluble and Urea-soluble brain extracts (40 *μ*g protein) of both mouse types were prepared as described in Section 2. These extracts were then incubated in the presence of additional brain-matched Tris-soluble extract (10 *μ*g protein, serving as a source for kinases), 25 mM Tris (pH 7.4), protease inhibitor cocktail (no-EDTA) (Roche), 1 mM CaCl_2_, 10 mM MgCl_2_, and 16.7 *μ*M [*γ*-^32^P]-ATP for 3 minutes at room temperature in a final volume of 50 *μ*L. Endogenous phosphorylation was stopped by addition of 10 mM EDTA, 10 mM EGTA, 1 mM cold ATP and was immediately placed on ice. Then, the samples were subjected to an immunoprecipitation by anti-*α*-synuclein antibodies or anti-MetO antibodies as described in Section 2. Thereafter, equal protein amounts of the immunoprecipitants were subjected to an SDS-gel electrophoresis (4–20%) followed by exposure of the gel to an X-ray film. Lanes 1, 3, 5, and 7 represent Tris-soluble fractions, and lanes 2, 4, 6, and 8 represent urea-soluble fractions. Syn: *α*-synuclein; ab: antibodies; kDA: molecular mass markers in kilo-Dalton. The detected band following the immunoprecipitation by anti-MetO antibodies was also denoted in the text as MetO-15.

